# The African Health Initiative’s Role in Advancing the Use of Embedded Implementation Research for Health Systems Strengthening

**DOI:** 10.9745/GHSP-D-22-00318

**Published:** 2022-09-15

**Authors:** Abdul Ghaffar, Livia Dal Zennaro, Nhan Tran

**Affiliations:** a Alliance for Health Policy and Systems Research, World Health Organization, Geneva, Switzerland.; b Department of the Social Determinants of Health, World Health Organization, Geneva, Switzerland.

## Abstract

The African Health Initiative has demonstrated the feasibility of changing the traditional knowledge generation paradigm by using an embedded implementation research approach to improve health systems’ performance and strengthen capacity for knowledge generation and use.

## Key Messages

- The embedded implementation research (EIR) approach involves engaging policy makers and decision makers at each stage from the design to the dissemination of research and increases the likelihood of the use of research evidence to better inform policy and management decisions.
- The African Health Initiative is an example of a public enterprise that has demonstrated success in developing domestic research capacity and strengthening health systems in sub-Saharan Africa by integrating the EIR approach into its projects.
- Further efforts and investments are needed to increase the sustainability and scalability of the EIR approach to improve the performance of national health systems.

Embedded implementation research (EIR) is an approach to integrating knowledge generation and decision making about policy and program implementation that has been conceptualized and promoted by the Alliance for Health Policy and Systems Research (AHPSR) since 2012. The understanding and use of EIR as an approach to address knowledge gaps in health systems is gaining traction within the global health community. The African Health Initiative (AHI) has built on the EIR approach and demonstrated notable success in pursuing the twin agendas of health systems strengthening and research capacity strengthening. Since its inception, the AHI has produced more than 350 publications; trained more than 3,000 researchers, decision makers, and health workers; and strengthened the institutional capacities of more than 300 health facilities and research institutions across Africa.^1^ In addition to having influenced the policies and practices of many countries, this work has also helped to shape the thinking of researchers, policy makers, and development partners on the use of EIR as a means of health systems strengthening.^2^ This longstanding collaboration has engendered extensive learning about the effectiveness of the EIR approach and its contributions to improving the use of knowledge to strengthen health systems. In this commentary, we trace the history and growth of EIR and encapsulate its key learnings through the AHI’s work.

## HISTORY AND EVOLUTION OF EIR

In December 2009, development experts, multilateral funders, national stakeholders, and researchers convened by the Norwegian Development Agency met to discuss the challenges and limited progress toward attaining the health-related Millennium Development Goals. The group cited a failure to implement proven effective interventions to reduce child and maternal mortality and highlighted the urgent need to invest in research to achieve better impact. After this meeting, in 2010, the Implementation Research Platform was established within the AHPSR to support and build capacity among national stakeholders to use implementation research as a means of attaining the Millennium Development Goals.^3^ This coincided with increasing recognition within the health sector of the relevance and importance of implementation research as a critical component of development initiatives.

One salient example of this work in low- and middle-income countries was the AHI. In 2009, the Doris Duke Charitable Foundation’s (DDCF) AHI funded 5 partnerships between policy decision makers in Ministries of Health, host country– and U.S.-based academic institutions, and local health system implementation leaders in Ghana, Mozambique, Rwanda, Tanzania, and Zambia to design, implement, and study multifaceted interventions specific to local contexts to strengthen systems to improve primary care and, thereby, demonstrate effective and feasible strategies for overcoming the globally recognized “know-do” gap.^4^ Intrinsic to the AHI strategy, across countries, was the integration of implementation research in large-scale, public-sector primary health care programs as a means for promoting evidence use, collaborative learning, and adaptation in the systems strengthening process.^5^^,^^6^ The Table summarizes the evolution of EIR since the AHI’s inception.

Intrinsic to the AHI strategy was the integration of implementation research in large-scale, public-sector primary health care programs to promote evidence use, collaborative learning, and adaptation in the systems strengthening process.

**TABLE.** Evolution of Embedded Implementation Research

**Date**	**Critical Event**
2009	AHI funded 5 countries to do HSS using an integrated and multistakeholder approach.
2009	Experts and stakeholders convened by Norwegian Development Agency met to address challenges in achieving Millennium Development Goals and knowledge gaps.
2010	Held Inaugural Health Systems Research symposium and established Implementation Research Platform within WHO.AHI convened senior leadership meeting to share emerging lessons.
2012	Launched WHO Strategy on Health Policy and Systems Research: Changing Mindsets.
2013	Published first AHI supplement on HSS and EIR.
2013	Implementation Research Platform released first call for implementer-led research.
2014	Launched Implementation Research and Delivery Research initiative by AHPSR, World Bank, and U.S. Agency for International Development.Launched EIR initiatives in WHO Eastern Mediterranean Regional Office and PAHO.
2015	Held joint EIR call between AHPSR, UNICEF, and Gavi.
2015	AHPSR met with Doris Duke Charitable Foundation leadership to discuss AHI Phase 2.First AHI-supported PhD students in EIR graduated.
2016	Launched Strengthening Capacity for Implementation Research initiative by AHPSR.
2017	Published AHI second supplement documenting results of EIR including research capacity building success.Funded AHI 2.0 in Mozambique, Ghana, and Ethiopia including ongoing focus on EIR approach as target for strengthening health systems and ongoing research capacity building.
2018	Launched EIR call in Pakistan on immunization by AHPSR, UNICEF, and Gavi.
2019	Launched EIR call on the Sustainable Development Goals in collaboration with PAHO and WHO Special Programme for Research and Training in Tropical Diseases.
2020	EIR identified in special supplement of Health Policy and Planning on Innovations in Implementation Research as a key innovation signaling the coming-of-age of implementation research field.
2020	Launched EIR Accelerator Project in Ethiopia, Uganda, Indonesia, and Nigeria, with AHPSR supporting Gavi programs through implementation research in each of these countries.
2021	Launched Compassionate and Respectful Health Care Services Ethiopia on health workforce and health services by AHPSR, WHO Health Workforce Department, and WHO Country Office Ethiopia.AHI grantees held final meeting in Accra, Ghana.
2022	AHPSR’s MAINSTREAM initiative embedded implementation research into Gavi’s learning strategy for its new strategic plan period (Gavi 5.0)

Abbreviations: AHI, African Health Initiative; AHPSR, Alliance for Health Policy and Systems Research; EIR, embedded implementation research; HSS, health systems strengthening; PAHO, Pan American Health Organization; WHO, World Health Organization.

In 2012, the World Health Organization (WHO) published its *Strategy on Health Policy and Systems Research: Changing Mindsets*.^7^ Developed by the AHPSR, this strategy called for embedding research into policies and program implementation; this was the genesis of the use of EIR by the AHPSR and WHO. To operationalize this concept, the Implementation Research Platform grant schemes were restructured. Whereas previous research grants were awarded to research investigators, a new scheme was developed to target research funding to practitioners and policy actors. Later referred to as implementer-led research, this scheme fostered leadership and ownership of the research by implementers as the principal investigators, with support from researchers on the technical aspects of the design and methodology.

The breadth of experience in EIR continued to expand, with its benefits accruing to major health schemes and programs that made use of the approach. In Ethiopia, the University of Gondar’s collaborations on EIR with the Ministry of Health have been instrumental in shaping district-level and national policies for immunization and the health workforce, respectively.^8^ In India, the National Health Authority engaged several leading Indian research institutions in conducting EIR in relation to India’s National Health Insurance Scheme. In these instances, workshops focused on the interpretation and dissemination of the research findings, which have been critical in helping to adapt and improve program delivery.^9^^–^^11^ In Argentina, EIR resulted in the development of new monitoring indicators to improve the implementation of the perinatal health policy. In Chile, research findings led to the establishment of a training program on reproductive rights that targeted municipal health facilities.^12^

This innovation in the design of implementation research funding became a game changer. After the initial scheme was launched by the Implementation Research Platform in 2013, additional implementer-led research grant schemes were launched in collaboration with UNICEF and Gavi, the Vaccine Alliance. This approach was subsequently embraced by the WHO Regional Offices, especially the Eastern Mediterranean Regional Office and Pan American Health Organization, as well as the World Bank and U.S. Agency for International Development, as part of their Implementation Research and Delivery Science initiative launched in 2014 in collaboration with the AHPSR at the third Global Symposium for Health Systems Research. Since then, the AHPSR has supported more than 140 studies across 45 countries.

Coinciding with this work by the AHPSR, although they were not specifically framed as EIR initiatives, the AHI projects were actively designing and conducting implementation research projects that were embedded within existing health systems strengthening initiatives. To guide this, all AHI partnerships included research conducted in primary health care systems that was aimed at helping implementers address major policy implementation challenges, drawing upon the WHO health system building blocks framework as a guide for adapting holistic, integrated strategies for solving problems that cut across traditional programming silos. Furthermore, the AHI partnerships adhered to a common evaluation framework that sought data that could elucidate the causal chain between HSS inputs and activities and health outcomes, including contextual factors and implementation strength as potential determinants.^13^ The work adapted and employed this framework to the effect of understanding processes and outcomes of a wide range of HSS strategies.

For example, the AHI partnership in Ghana designed and assessed a strategy aimed at accelerating the scale-up of longstanding primary health care policy; the Tanzania team developed operational guidance on establishing a national community health worker program and evaluating its feasibility and health impact; and, in Mozambique, the AHI partnership developed and evaluated strategies to strengthen district health systems to implement decentralization policies.^14^^–^^16^ The work also focused on building the next generation of researchers embedded in government institutions and point-of-care settings. This was achieved through cross-cutting investments in Master’s and Doctoral scholarships for local researchers, strengthening research administrative systems and infrastructure, conducting in-service trainings in data use, strengthening mentorship networks, and routinely convening multidisciplinary fora that encouraged stakeholders to increase the demand for and uptake of the research results.^17^

An important turning point in the evolution of this work occurred in September 2015, when the DDCF and the AHPSR met to design Phase 2 of the AHI work and discovered a strong alignment of interests and similarities between the AHI methods and the EIR approach used by the AHPSR. The emphasis on research, evidence-informed policy development, and focus on supporting decision makers at all levels of the health system to scale effective strategies that arose from the first phase of partnerships was strongly aligned with the principles of the EIR approach. As a result, in 2016, the EIR approach was incorporated into the design of the second phase of the AHI. The AHI’s adoption of EIR made the DDCF the biggest investor in EIR, and, with the AHPSR’s support, the AHI became an important initiative to engage with development partners, national governments, and the implementation research/health systems research community to promote and use EIR.

During this time, the AHPSR continued to strengthen its focus on EIR, with additional calls for research targeting implementers/decision makers and programs to develop the capacity for EIR in low- and middle-income countries. This included the establishment of regional hubs that provided trainings and local research grants using the EIR approach. At the same time, the AHPSR worked with research and policy institutions to further build the field of EIR and contribute to the global body of knowledge.^18^^–^^25^

In 2017, the AHI published a journal supplement that provided some of the first evidence on the feasibility of having decision makers drive implementation research studies and the effectiveness of the EIR approach in improving program implementation and health outcomes.^26^ The supplement described the implementation approaches and outcomes in cross-cutting areas that are critical for accelerating and measuring HSS, including increasing data quality and use, quality of care improvement, supervision and mentoring, and research capacity strengthening, as well as challenges in approaches to evaluating HSS. The articles represented progress in the wider field of EIR, and the results they described reflected how teams collectively learned and addressed implementation challenges in specific contexts. Moreover, this supplement and the previously mentioned work sponsored and supported by the AHPSR contributed to a global evidence base that has helped inform policy makers, managers, and implementation researchers on how to choose interventions to strengthen primary health care, adapt their implementation in different environments, and evaluate implementation processes and outcomes.

## KEY LEARNINGS FROM USING EIR

The generation and use of contextually relevant evidence to inform the implementation of policies and programs has been and continues to be an important challenge in the attainment of global health goals. Despite many efforts to increase the uptake of evidence through researcher-driven initiatives, there has only been limited progress.^27^ The conventional paradigm of a researcher-driven evidence generation process perpetuates a power imbalance in the prioritization, planning, generation, and use of knowledge that fails to bridge the worlds of policy and research.^28^ The use of EIR in AHPSR-supported initiatives and AHI-funded projects has demonstrated the feasibility of changing the traditional knowledge generation paradigm resulting in greater collaboration and a balance of power between those that generate and those that use knowledge.^29^ Some of the most significant learnings from this work over the years can be summarized as follows.

1. EIR has been shown to be an effective means of improving program and policy implementation and can be applied to a range of health issues, including maternal, newborn, and child health and immunization, as well as to several health targets within the Sustainable Development Goals.
2. Through the AHI, EIR has shown promise in also serving as a tool to help strengthen health systems performance and as a means of building capacity for knowledge generation and use.
3. The engagement of multiple stakeholders, particularly decision makers, in setting research agendas, designing, and building solutions is critical to ensure that EIR is responsive to implementers’ and communities’ felt needs and evaluation methods are fit for the decision context and purpose as part of the EIR process.
4. Multilevel feedback loops to assess programs and inform decision making are needed as part of the iterative implementation process.
5. EIR directly responds to the problems/questions of implementers/program managers and increases the relevance and use of the evidence generated.

The use of EIR in AHPSR-supported initiatives and AHI-funded projects has demonstrated the feasibility of changing the traditional knowledge generation paradigm resulting in greater collaboration and a balance of power between those that generate and those that use knowledge.

Despite encouraging outcomes, the EIR approach also faces a number of barriers and challenges. A significant one has been the issue of time and political processes.^12^ During the research process, the time frames and needs of researchers and decision makers may not often align, perpetuating biases among these groups, which may already be present. Reconciling these differences and biases can be addressed by sharing and showing examples where EIR has significantly improved the implementation of activities and programs in different settings.

## CONCLUSION

The failure to implement remains one of the key barriers to progress in global health. The EIR approach has been developed to address this critical challenge. During the last 10 years, EIR has been used successfully to improve the use of knowledge in decision making at different levels within health systems, resulting in tangible outcomes that have been documented in numerous publications.^8^^–^^12^ The EIR approach, as demonstrated by the AHI, has shown its potential for strengthening the learning capacity of health systems and improving health systems’ performance. While further evaluations of the EIR approach are needed, there is clearly strong support for this approach to contribute significantly to improving policy and program implementation. Achieving impact at scale will demand replication of experiences like the AHI. Governments, experts, and donors should also embrace the EIR approach as a central element of their development agenda.

The AHI demonstrated notable potential and DDCF’s investments are proof of how governments can support health systems and research capacity strengthening by adopting the EIR approach. Now, the challenge is to ensure that EIR is fully integrated into health service delivery and not funded as stand-alone projects. We conclude on an optimistic note and hope that the investments made to date by the DDCF, AHPSR, and other partners to strengthen individual and institutional capacities will serve as a seed for this approach to grow.

## Notes

**Peer Reviewed**

## References

[1] African Health Initiative. Doris Duke Charitable Foundation. Accessed August 25, 2022. https://www.ddcf.org/funding-areas/african-health-initiative/

[2] Ghaffar A Langlois EV Rasanathan K Peterson S Adedokun L Tran NT. Strengthening health systems through embedded research. Bull World Health Organ. 2017;95(2):87. 10.2471/BLT.16.189126. 28250505 PMC5327943

[3] World Health Organization (WHO). *Implementation Research in Health. A Practical Guide*. WHO; 2013. Accessed August 25, 2022. https://apps.who.int/iris/bitstream/handle/10665/91758/9789241506212_eng.pdf

[4] Pakenham-Walsh N. Learning from one another to bridge the “know-do gap”. BMJ. 2004;329(7475):1189. 10.1136/bmj.329.7475.1189

[5] Binagwaho A Nutt CT Uwaliraye P Wagner CM Nyemazi JP. Taking health systems research to the district level: a new approach to accelerate progress in global health. BMC Health Serv Res. 2013;13(Suppl 2):S11. 10.1186/1472-6963-13-S2-S11. 23819798 PMC3668257

[6] Bassett MT Gallin EK Adedokun L Toner C. From the ground up: strengthening health systems at district level. BMC Health Serv Res. 2013;13(Suppl 2):S2. 10.1186/1472-6963-13-S2-S2. 23819479 PMC3668220

[7] World Health Organization (WHO). *Strategy on Health Policy and Systems Research: Changing Mindsets*. WHO;2012. Accessed August 25, 2022. https://apo.who.int/publications/i/item/2015-07-15-who-strategy-on-health-policy-and-systems-research

[8] Tilahun B Zelalem M Mekonnen ZA. Embedding implementation research to strengthen efforts towards improving primary health care in resource limited settings. Ethiop J Health Dev. 2021;35:1–3. Accessed August 25, 2022. https://ejhd.org/index.php/ejhd/article/view/4646/1730

[9] Furtado KM Raza A Mathur D Vaz N Agrawal R Shroff ZC. The trust and insurance models of healthcare purchasing in the Ayushman Bharat Pradhan Mantri Jan Arogya Yojana in India: early findings from case studies of two states. BMC Health Serv Res. 2022;22(1):1056. 10.1186/s12913-022-08407-2. 35982425 PMC9389741

[10] Saxena A Trivedi M Shroff ZC Sharma M. Improving hospital-based processes for effective implementation of Government funded health insurance schemes: evidence from early implementation of PM-JAY in India. BMC Health Serv Res. 2022;22(1):73. 10.1186/s12913-021-07448-3. 35031024 PMC8760668

[11] Trivedi M Saxena A Shroff Z Sharma M. Experiences and challenges in accessing hospitalization in a government-funded health insurance scheme: evidence from early implementation of Pradhan Mantri Jan Aarogya Yojana (PM-JAY) in India. PLoS One. 2022;17(5):e0266798. 10.1371/journal.pone.0266798. 35552557 PMC9098065

[12] Langlois EV Mancuso A Elias V Reveiz L. Embedding implementation research to enhance health policy and systems: a multi-country analysis from ten settings in Latin America and the Caribbean. Health Res Policy Sys. 2019;17(85). 10.1186/s12961-019-0484-4. 31615511 PMC6794825

[13] Bryce J Requejo JH Moulton LH Ram M Black RE; Population Health Implementation and Training – Africa Health Initiative Data Collaborative. A common evaluation framework for the African Health Initiative. BMC Health Serv Res. 2013;13(Suppl 2):S10. 10.1186/1472-6963-13-S2-S10. 23819778 PMC3668298

[14] Awoonor-Williams JK Bawah AA Nyonator FK. The Ghana essential health interventions program: a plausibility trial of the impact of health systems strengthening on maternal & child survival. BMC Health Serv Res. 2013;13(Suppl 2):S3. 10.1186/1472-6963-13-S2-S3. 23819518 PMC3668206

[15] Ramsey K Hingora A Kante M. The Tanzania Connect Project: a cluster-randomized trial of the child survival impact of adding paid community health workers to an existing facility-focused health system. BMC Health Serv Res. 2013;13(Suppl 2):S6. 10.1186/1472-6963-13-S2-S6. 23819587 PMC3668255

[16] Sherr K Cuembelo F Michel C. Strengthening integrated primary health care in Sofala, Mozambique. BMC Health Serv Res. 2013;13(Suppl 2):S4. 10.1186/1472-6963-13-S2-S4. 23819552 PMC3668215

[17] Hedt-Gauthier BL Chilengi R Jackson E; with input from the AHI PHIT Partnership Collaborative. Research capacity building integrated into PHIT projects: leveraging research and research funding to build national capacity. BMC Health Serv Res. 2017;17(Suppl 3):825. 10.1186/s12913-017-2657-6. 29297405 PMC5763288

[18] Compassionate and Respectful Care in Ethiopia. Ethiopian J Health Biomedical Sci. 2022;12(1):1–110. 10.20372/ejhbs.v12i1SpecialIssue

[19] Using Embedded Implementation Research to Improve Immunization Services and Expand Coverage in Ethiopia. Ethiopian J Health Dev. 2021;35(3). Accessed August 25, 2022. https://ejhd.org/index.php/ejhd/issue/view/147

[20] Pakistan Embedded Implementation Research for Immunization Initiative. *J Glob Health*. 2021;11. Accessed August 25, 2022. https://jogh.org/category/jogh/jogh-collections/pakistan-embedded-implementation-research-for-immunisation-initiative/

[21] Embedded Implementation Research for the Sustainable Development Goals. *Rev Panam Salud Publica*. 2021;45. Accessed August 25, 2022. https://www.paho.org/journal/en/special-issues/embedded-implementation-research-sustainable-development-goals

[22] Decision Maker Led Implementation Research on Immunization. Health Res Policy Syst. 2021;19(Suppl 2). Accessed August 25, 2022. https://health-policy-systems.biomedcentral.com/articles/supplements/volume-19-supplement-2 10.1186/s12961-021-00720-2 PMC8356372 34380520

[23] Innovations in Implementation Research in Low- and Middle-Income Countries. Health Policy Plan. 2020;35(Suppl 2). Accessed August 25, 2022. https://academic.oup.com/heapol/issue/35/Supplement_2 10.1093/heapol/czaa120 PMC7646729 33156931

[24] Addressing Implementation Challenges for Maternal, Newborn and Child Health Interventions. Acta Paediatr. 2018;107(S471):1–88. Accessed August 25, 2022. https://onlinelibrary.wiley.com/toc/16512227/2018/107/S471 10.1111/apa.14641 30570798

[25] Implementation Research in the Americas. *Rev Panam Salud Publica*. 2017;41. Accessed August 25, 2022. https://iris.paho.org/browse?value=Rev%20Panam%20Salud%20Publica;41,%20abr.%202017&type=serie

[26] Implementation Science as an Essential Driver for Sustainable Health Systems Strengthening Interventions: Lessons Learned Across the Five-Country African Health Initiative. BMC Health Serv Res. 2017;17(Suppl 3). Accessed August 25, 2022. https://bmchealthservres.biomedcentral.com/articles/supplements/volume-17-supplement-3

[27] Greenhalgh Trisha Russell Jill. Evidence-based policymaking: a critique. Perspect Biol Med. 2009;52(2):304–318. 10.1353/pbm.0.0085. 19395827

[28] EVIPNet: Evidence-informed Policy Network. World Health Organization. Accessed August 25, 2022. https://www.who.int/initiatives/evidence-informed-policy-network

[29] Redman S Greenhalgh T Adedokun L Staniszewska S Denegri S; Co-production of Knowledge Collection Steering Committee. Co-production of knowledge: the future. BMJ. 2021;372:n434. 10.1136/bmj.n434. 33593753 PMC7884902

